# Scoping literature review on the Learning Organisation concept as applied to the health system

**DOI:** 10.1186/s12961-017-0176-x

**Published:** 2017-03-01

**Authors:** E. Akhnif, J. Macq, M.O Idrissi Fakhreddine, B. Meessen

**Affiliations:** 1 Ministry of Health, Rabat, Morocco; 2 Université Catholique de Louvain, Louvain-la-Neuve, Brussels, Belgium; 3 Institute of Tropical Medicine, Antwerp, Belgium; 4 Ecole National de Santé Publique, Ecole Nationale de Santé Publique, Rabat, Morocco; 5 Community of Practice “Performance Based Financing”, Antwerp, Belgium

**Keywords:** Learning organisation, Health, Health systems, Universal health coverage

## Abstract

**ᅟ:**

There is growing interest in the use of the management concept of a ‘learning organisation’. The objective of this review is to explore work undertaken towards the application of this concept to the health sector in general and to reach the goal of universal health coverage in particular. Of interest are the exploration of evaluation frameworks and their application in health.

**Method:**

We used a scoping literature review based on the York methodology. We conducted an online search using selected keywords on some of the main databases on health science, selected websites and main reference books on learning organisations. We restricted the focus of our search on sources in the English language only. Inclusive and exclusive criteria were applied to arrive at a final list of articles, from which information was extracted and then selected and inserted in a chart.

**Results:**

We identified 263 articles and other documents from our search. From these, 50 articles were selected for a full analysis and 27 articles were used for the summary. The majority of the articles concerned hospital settings (15 articles, 55%). Seven articles (25%) were related to the application of the concept to the health centre setting. Four articles discussed the application of the concept to the health system (14%). Most of the applications involved high-income countries (21 articles, 78%), with only one article being related to a low-income country. We found 13 different frameworks that were applied to different health organisations.

**Conclusions:**

The scoping review allowed us to assess applications of the learning organisation concept to the health sector to date. Such applications are still rare, but are increasingly being used. There is no uniform framework thus far, but convergence as for the dimensions that matter is increasing. Many methodological questions remain unanswered. We also identified a gap in terms of the use of this concept in low- and middle-income countries and to the health system as a whole.

## Introduction and background

The learning organisation (LO) is a relatively a new concept that owes a lot to *The Fifth Discipline*, the seminal publication by Senge [1]. The early works of Senge [1] and Garvin [2] gave rise to the first definitions and features of a LO. These authors, and others, also proposed frameworks and tools that made the LO concept operational and less abstract [3–6].

A lot of this early conceptualisation work was inspired by the analysis of the experiences of major private companies, which realised the importance of investing in knowledge and learning to reach higher levels of creativity and innovation. Ensuring that a company has the attributes of a LO is considered in today’s fast changing environment as a source of competitive advantage. Therefore, the LO concept is embedded in the minds and visions of managers of leading companies, as well as smaller ones, worldwide.

The research agenda has followed this dynamic with interest, with researchers exploring several directions, including analysing the relationship between adopting a LO model and the financial performance of firms [7, 8]. For non-profit organisations, Wetherington et al. [9], in 2013, led a study that confirmed the relationships found in earlier studies between the dimensions of the LOs and financial, knowledge and mission performance. The lenses of the LO concept have also been applied to the public sector; for instance, Rose et al. [10] examined the relationship among the LO variables and other organisational variables by using a sample of public service managers in Malaysia. Organisational learning was found to be positively related to organisational commitment, job satisfaction and work performance [10]. In this review, public as well as private, for- and not-for-profit organisations are included. A systematic review conducted by Rashman et al. [11] examined aspects of organisational learning in public organisations and concluded that frameworks for explaining processes of organisational learning at different levels need to be sufficiently dynamic and complex to effectively accommodate public organisations.

Learning from the private and non-health sectors that have applied and succeeded in getting some positive results from reorganising according to LO models should lead us to ask what those companies and a health system might have in common.^1^ Much like commercial companies, health systems face challenges related to patient satisfaction, improvement of health at individual and population level, and the scarcity of resources. These too require a creative approach to designing policies that maximise outcomes with the limited available resources. The health system also operates in a dynamic environment, be it epidemiological, demographical, financial or political.

Research is a key learning pathway for the health sector. The use of research is actually embedded in the concept of LO as one important source of knowledge. The types of knowledge essential to improve the performance, and more specifically, the learning capacities of an organisation or a system may however be broader than the ones traditionally generated by scientific work. The type of research that is promoted by the LO concept is linked to action, for example, the research can provide tools like the collective resolution of problems and the documentation of experiences to make stored implicit knowledge explicit. With well-organised knowledge translation mechanisms, research can play an important role to bridge the gap between knowledge produced from the action and the decision-making process.

There are many reasons for health sector actors to pay more attention to the concept of LOs, and we believe that the emerging global movement towards universal health coverage (UHC) only increases the need for better ‘learning’ health systems. UHC is defined as the capacity to provide all people with access to needed health services (including prevention, promotion, treatment and rehabilitation) of sufficient quality to be effective, while also ensuring that the use of these services does not expose the user to financial hardship [12]. The United Nations General Assembly resolution adopted on December 12, 2012, urged governments across the world to move towards UHC [13]. There is an emerging body of literature on the paths to UHC. For instance, a recent study looked at 24 countries [14] and highlighted the diversity of paths to UHC, both in terms of strategic choices and results. In fact, for any country, the road to UHC is inextricably linked to the complex process by which policy decisions take place. The quest to make progress towards UHC obliges countries to undertake major transformations to their health systems, especially in the area of healthcare financing. The technical nature of the reforms generates a fair amount of confusion, including the flawed perception that UHC is about introducing some specific arrangements such as social health insurance. In reality, each country has to find and follow its own path to UHC, starting from its current situation [15].

If there is one universal recommendation for UHC, it is that countries should be willing to engage in permanent learning [15]. Innovation, creativity and the capacity to learn from one’s own experience, but also from experiences of other countries, become the key resources for development. Progressing towards UHC is mainly about making strategic choices, while simultaneously dealing with the complexity of the health system. This is why integrating systemic learning within health system organisations becomes the only path for countries to develop contextualised strategies inspired by internal and external experiences.

Over the last decade, a growing number of experts and global actors have recommended adaptive strategies to strengthening health systems [16, 17]. Strengthening health systems requires dealing with their complexity and taking into account not only their components but also their complex interrelations, and adopting new ways of thinking to close the knowledge–action gap, where each innovation in health systems constitutes a learning opportunity [18]. Within this context, some interest for the LO model has emerged, but mostly regarding the application of the model to specific healthcare organisations; there is a need to identify and analyse the results of these applications to date.

The main purpose of this paper is to identify and assess the body of literature on the application of the concept of a LO to the health system. We are interested to know more about the frameworks used in assessing the LO in the health system, the methodological approaches used and instances of applying the concept to move towards UHC. To achieve these objectives, we performed a scoping review in order to gain a clear idea about how previous research has applied the concept of LO, and to which aspects or entities of the health system, using what analytical frameworks. The result of this research will add knowledge to the potential application of the LO concept to the health system by highlighting lessons, challenges and gaps. An additional complementary objective for us is to identify frameworks that can inform our future empirical work on the role of systemic learning capacities for low- and middle-income countries (LMICs) in their path to achieving UHC goals.

Our hypothesis is that the LO might have real analytical and prescriptive power, on the condition that it is adjusted to health system realities and particularly to the process of change on the path to UHC.

Therefore, our review focuses on identifying the applications of a LO to the health sector, and the tools and frameworks used so far for this.

## Method

We undertook a scoping review of the literature. We followed the classical steps required for a scoping review of the literature [19], namely identifying the research question, identifying relevant studies, study selection, charting the data, and collating, summarising and reporting the results.

### Identifying the research question

Taking into account the limited resources we had to perform this study, we adopted a pragmatic approach in order to get good results without unnecessarily broadening the scope of the review. Instead of exploring the history of the LO concept, its definitions and applications outside the health sector (work we have done in parallel, but which is not reported in this article), we decided to focus on assessing its use in the health sector. This choice was supported by an initial, quick research that highlighted the existence of literature reviews that have treated the LO and the organisational learning concepts in general [11]. During this preliminary non-systematic search, we found very little on the use of LO in the health sector, while the business sector has benefited from sufficient research in this area. Additionally, many articles examined organisational learning in general and the knowledge management, but very little was specific to LO, which is quite a recent concept, emerging in 1990 [1]. This research aims at assessing and analysing health organisations through the lens of a LO.

Therefore, we defined our main research question as follows: “How is the LO concept applied to the health sector, and more specifically in the context of the UHC agenda?”

The sub-questions related to this research question are presented as follows:

1. How is the LO concept applied to the health sector, in particular in assessing the existence of its characteristics or evaluating implemented experiments at different levels of the health system?
2. What are the frameworks used to assess the LO concept in the health sector (What dimensions are adopted? For what objective?)?
3. What practical approaches and empirical methods are used to assess LO in the health sector (documentation review, action research, surveys, etc.)?
4. What are the main findings of each application of a LO?
5. Are there any applied studies that have explored the link between the LO and progress towards UHC?

The hypotheses of the research are as follows:

- The LO model will contribute to the development of health strategies and policies by promoting organisational learning.
- Organisational learning cannot be maximised unless a LO structure is in place to make sure that the learning is promoted and used in the decision-making process.
- The LO concept could help LMICs in structuring their own capacity to design, adapt and improve strategies to move towards UHC.

### Identifying relevant studies

#### Electronic databases and identification of relevant studies

First, we restricted our search to some of the main databases in the health field, namely PubMed and the Web of Science (formerly ISI Web of knowledge). We also used the Google Scholar web search engine, by limiting the search to the health field. The focus of the search was mainly on electronic articles. Indeed, we worked on the assumption that most of the articles on LO would be available in an electronic format online, as the history of the LO concept is relatively recent. We also, therefore, did not restrict our search criterion by any date or time. Our study protocol was completed, and the searching process finalised, by October 1, 2015. One co-author (MOIF) had already participated in several scoping reviews in other fields and helped the first author to define the chain of keywords. The following research equations were used for each search engine: “learning organisation” and “health”; “learning organisation” and “health system”; “learning organisation” and “health”; “learning organisation” and “universal health coverage”; “learning organisation and “universal health coverage”. All literature database searches were restricted to the English language.

#### Website searching

In order to be more comprehensive in our search, we used the criterion to identify some main websites which focus on health and health policy, and some features of the LO concept. As the interest was in UHC and its links with the LO, we chose the WHO website because, in recent years, the WHO has been promoting UHC and health system strengthening. We also included the World Bank website for the same reasons.

#### Other literature sources outside the health sector

As mentioned earlier, the objective of this review was to inform our future empirical work. To complete the research on frameworks we selected three reference books [3–5] on the subject (for almost all the recent non-health sector research). The selection of those three books was based on the number of citations provided by Google Scholar. We also used other articles that are not covered by the keyword search to enrich the introduction to the subject, but these were not used in the summary.

#### Key informants and experts in the field

The research protocol was presented and discussed in a meeting gathering experts and professors at the Institute of Tropical Medicine. Their comments and suggestions were included in the final version of the research protocol.

### Article selection

**Fig. 1:**
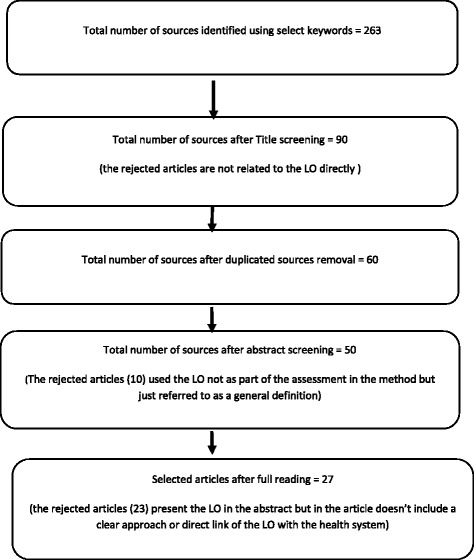
Stages of the selection process of the scoping review

The selection of articles was performed in several stages. The first stage of the selection was based on the title, so the inclusion criteria were set to include only sources with clear indication about the LO concept and a mention of the health field. After title screening, we analysed the remaining articles, identified duplicated sources and merged different results into one database using Reference Manager. However, we also verified our hypothesis by randomly checking the rejected articles and found they did not contain models or assessment tools on LO. We then undertook a review of the abstracts to identify the sources with a clear mention and analysis of the LO as a concept and framework to exclude those referring to the subject without deep analysis or application. A final list of detailed reading was thus formulated. Further research using references cited in the selected sources was performed, particularly of the ones that analysed LO models. In the last stage, complete (full length) versions of the selected, relevant articles were read and reviewed. For this final stage, the inclusion and exclusion criteria were as follows: (1) the source has a clear mention of the LO concept with reference to a definition of a framework, (2) there is a clear assessment approach in the method of a health organisation, (3) the article is directly related to the health sector. The review was not systematically conducted by more than one reviewer, as our criteria are straightforward, although we did discuss including some articles when ambiguity arose. Figure 1 presents the different stages of the selection.

### Charting the data

Guided by the scoping methodology described above, the charting of data refers to the stage during which information was extracted from the selected sources (review of full articles).

The content of the charting tool includes: (1) the setting (the country where the research was conducted); (2) the level of application of the concept (health centre, hospital, the whole system, etc.); (3) the objective of the research; (4) the adopted definition of the LO; (5) the reference framework used; (6) the methodological approach and tools used for the study; (7) the link with the health system as a whole or with UHC; and (8) the main findings.

## Results

The results will be presented according to a narrative synthesis with a focus on the application by levels of the health system as well as the main frameworks that were used.

### Collating, summarising and reporting the results

We initially retrieved 263 papers, out of which 50 were selected for their relevance to the research objectives. These 50 articles, 27 articles that directly analyse the application of the LO concept and discuss the applied frameworks were included in our summary. The remaining 23 articles treated the LO without deep analysis or use of frameworks and were therefore excluded; for the majority of these articles, the LO was referred to without using a clear approach to its analysis.

#### The settings

We analysed the settings related to our selected articles. The analysis by the level of income of countries indicated that 21 articles (78%) dealt with research conducted in high-income countries, five with research conducted in middle-income countries (18%), and only one article was related to a case in a low-income country (Nepal) (Table 1).

**Table 1:** Country distribution of studies on learning organisations conducted

Country	Number of articles	%
United States of America	6	22%
United Kingdom	8	30%
Turkey	2	7%
Iran	2	7%
Australia	3	11%
Nepal	1	4%
Netherlands	1	4%
Oman	1	4%
Portugal	1	4%
South Korea	1	4%
Taiwan	1	4%
Total	27	100%

#### Year of publication of the selected articles

To analyse the possible growing interest for the subject we analysed the distribution of the included articles over three main periods of publication. We counted 6 articles written in the period between 1998 and 2005, 9 for the period between 2006 and 2010, and 12 written in the period between 2011 and 2015. We acknowledge here that the number of articles is limited, but these results indicate that the LO concept is gaining interest among health sector analysts. The future will tell whether this was just a flash in the pan or a reflection of the early stage of a strong development.

#### Application of the LO by level of the health system

The LO concept can be applied to different organisational units. Our scoping review showed that the majority of articles were conducted in the hospital setting (15 articles, 55%), the second context to which the LO concept was applied was that of health centres (7 articles, 25%), and four articles discussed the application of the concept for the whole system (14%). The rest of the articles discussed the education process in medical schools or in health organisations in general.

We have paid a closer look to those applications of the LO concept in the health sector which use various frameworks and serve different objectives. The summary of our research is organised according to the use of the concept by level of the health system, and we try to capture the main frameworks that were used.

#### The health centres, primary healthcare level

In their article, Somunoglu et al. [20] analysed the perceptions of a LO application by workers at an oral and dental health centre in a city in Turkey [15]. They used a model based on the four blocks [21] of (1) the ‘knowing organisation’, (2) the ‘understanding organisation’, (3) the ‘thinking organisation’, and (4) the ‘learning organisation’. They used a questionnaire with a scale from one to five and the researchers interviewed 193 health workers active at health centre level. They concluded that there was a weak understanding of the LO concept among staff of the oral and dental care centre and stated that, while their institutions used some of the applications of a LO, the process of becoming a LO was not fully completed.

In their study, Kelly et al. [22] assessed the ‘collective learning capacity for change’ by measuring the learning characteristics of a first-line services practice team in Scotland. Their approach was inspired by Senge’s definition [1], as well as by other authors [23, 24]. A questionnaire was developed to collect 62 concepts related to the LO with a scale from one to ten and was administered to 85 staff members from five practices. The feedback collected through the questionnaire was considered interesting. All the five practices had median scores weighted towards the positive end of the scale for LO characteristics.

In their article, Schilling et al. [25] evaluated the implementation of a six-block model of the LO in 36 health centres of Kaiser Permanente in the United States of America. The study analysed the experience of implementing LO characteristics as an action research that used different data, including performance data, to evaluate the implementation of the LO. The six blocks were (1) real-time sharing of meaningful performance data, (2) formal training in problem solving methodology, (3) workforce engagement and informal knowledge sharing, (4) leadership structures beliefs and behaviours, (5) internal and external benchmarking, and (6) technical knowledge sharing. They concluded that the LO has the capability to develop structures and processes that facilitate the acquisition and sharing of knowledge. The study concluded that the six blocks have enabled Kaiser Permanente to become a LO.

Birleson et al. [26] evaluated an experience of applying the LO concept in a child and adolescent mental health service in Australia. The approach taken was action research, and consisted of reviewing the original organisational aims, analysing the performance of the service, examining the quality of clinical care recorded in external reports, and analysing structured interviews with directors of all metropolitan hospitals about the performance of their organisations and the achievement of the aims of the LO set in 1996. Their model is based on the early work of Birleson [27]. They looked at the dimensions of (1) leadership, (2) organisational design, (3) work design, (4) perception, (5) information processing, (6) communication, and (7) motivational systems. The authors reported that the implementation of LO enabled enhanced service delivery and improved quality of mental health services, based on the analysis of different indicators.

Cantle et al. [28] tried to identify the characteristics of a ‘learning organisation’ in a fundholding General Practice in the United Kingdom. They took an ethnographic approach written up as a case study using the dimensions of (1) policy, (2) operations, (3) action, and (4) ideas, drawn from the work of Pedler [23]. They concluded that the case study analysed contains the characteristics of a LO. Another review conducted by O’Connor and Kotze [29] analysed two frameworks and programs being conducted in New South Wales to conclude that the LO concept provides a useful conceptual framework and tools for individuals and organisations to apply in developing knowledge and affecting change. They analysed two models, namely model 1 (achieve your intended purpose, maximise winning and minimise losing, suppress negative feelings, behave according to what you consider rational, advocate your position, evaluate your own thoughts and actions and those of others, attribute causes for whatever you are trying to understand) and model 2 (valid information, informed choice, vigilant monitoring of the implementation of choice in order to detect and correct error, premises are made explicit, inferences from premises are made explicit, conclusions are crafted in a manner that can be tested by logic that is independent of the actor). Bunniss et al. [30] conducted action research on LO in a general practice in the United Kingdom using Senge’s LO framework [1] to conclude that it is possible to support healthcare staff in learning together.

#### Hospital level

Leufven et al. [31] assessed the use of the dimensions of the LO questionnaire (DLOQ), initially developed by Watkins and Marsick [32], in one hospital in Nepal. The questionnaire was administered to 230 employees at all levels of the hospital, using a 6-point Likert scale (1 – almost never, 6 – almost always). They used a framework with the following blocks: (1) the individual level, which is composed of two dimensions of organisational learning, namely continuous learning, dialogue and inquiry; (2) the team or group level, which is reflected by team learning and collaboration; (3) the organisational level, which has two dimensions of organisational learning, namely embedded systems and empowerment; and (4) the global level, which consists of two dimensions of organisational learning, namely systems connection and strategic leadership. The authors concluded that the DLOQ could be used and applied in hospital settings in low-income countries.

Mohebbifar et al. [33] aimed to specify the LO profile in educational hospitals in Iran, based on the LO blocks of Marquardt [4], which are (1) people, (2) learning, (3) organisation, (4) knowledge, and (5) technology. They administered a questionnaire of 50 items to 530 staff in those hospitals; the hospitals of two universities were found to be far removed from the characteristics of LO.

The relationship of the characteristics of the LO to registered nurses’ beliefs regarding evidence-based practice in six acute care hospitals in the United States was analysed by Estrada et al. [34]. They used the DLOQ of Watkins and Marsick [30], with the six scales, and the following framework: (1) create continuous learning opportunities, (2) promote inquiry and dialogue, (3) encourage collaboration and team learning, (4) establish systems to capture and share learning, (5) empower people toward a collective vision, (6) connect the organisation to its environment, and (7) use leaders who model and support learning at the individual, team and organisational levels [35]. They administered the questionnaire to 594 respondents and the results showed that the nurses rated their organisations in the mid-range on the dimensions of LO and their perceptions of the LO were found to be significant regarding the importance of the LO dimensions as presented above.

Dias and Escoval [36] tried to provide an analytical understanding of hospitals as ‘learning organisations’ and the link between innovation and performance in Portugal. They used a survey with 80 questions that analysed organisational attributes, which are implicitly considered as an organisation’s ability to learn. They collected responses from 95 administrators from hospital boards. The study concluded that hospitals classified as ‘advanced learning organisations’ are five times more likely to be innovative as compared to those classified as ‘basic learning organisations’. Oudejans et al. [37] discussed the internal consistency and factor structure of a questionnaire measuring learning capacity based on Senge’s five disciplines theory of LO in the context of substance-abuse treatment centres in the Netherlands. They used a questionnaire of 44 items according to the five disciplines of Senge’s theory and five scales and they interviewed 293 employees from the outpatient department. The authors concluded that the proposed five-factor structure was confirmed in the LO questionnaire, with a six-level scale, which makes it useful to assess learning capacity in teams.

A study on tertiary medical hospitals in South Korea was conducted by Jeong et al. [38] to examine the effect of individual nurses’ use of the principles of LO on organisational effectiveness based on Senge’s model. They used four questionnaires: the LO scale, the Organisational Commitment Questionnaire (to assess nurses’ characteristics based on job and demography), the General Satisfaction scale (employees’ satisfaction based on six principal items) and the LO scale (23 items in five factors). The sample comprised of 629 nurses who had worked for more than 1 year as full-time employees in the general units of tertiary medical hospitals. The authors concluded that individual nurses’ use of the principles of LO was a good method for enhancing organisational effectiveness in a healthcare setting.

Ugurluoglu et al. [39] conducted a study on hospitals in Turkey to analyse the relation between LO dimensions and innovation in healthcare, inspired by Watkins [40], Garvin [41] and Senge [1]. They interviewed 243 hospital managers working at 250 Ministry of Health (public) hospitals and used the DLOQ with a six-point scale ranging from ‘almost never’ to ‘almost always’. The blocks of this model were (1) continuous learning and continuous learning opportunities; (2) inquiry and dialogue, a culture of questions, feedback and experimentation; (3) team learning, collaboration and collaborative skills, which support effective use of teams; (4) empowerment, namely the process to create and share a collective vision and have feedback from members regarding the difference between present and shared vision; (5) embedded system of collective efforts to establish and capture shared learning; (6) system connection; and (7) providing leadership to promote learning. Through the analysis of the scores, they concluded that LO practices appear to be important for healthcare organisations because they have the potential for contributing to innovation, organisational commitment and effectiveness.

Vassalou [42] tried to understand the role of the learning principles of the LO and identify the barriers to their application in United Kingdom and Greek hospitals through a comparative qualitative research. The study was based on a literature review approach and 22 semi-structured interviews (one hour on average) with members of the Board, general managers, and staff of the United Kingdom and the Greek health organisations for the analysis. His framework covered the principles of (1) mission and vision, (2) leadership, (3) transfer of knowledge, (4) teamwork and cooperation and (5) an experimenting culture, and had two foundations, namely organisational design and employee skills and competencies. The study concluded that healthcare organisations in both countries encounter certain common barriers to the building of a LO.

Rowley [43] used the following framework in an acute care public hospital in Victoria, Australia, as a case study of a descriptive research to document the improvement of the commitment and satisfaction of its staff with the hospital’s leadership and approach, using the principles of the LO. The research was based on the results of repeated surveys performed in 1999, 2002 and 2004. The blocks of the framework were (1) provide continuous learning opportunities, (2) use learning to reach their goals, (3) link individual performance with organisational performance, (4) foster inquiry and dialogue, (5) make it safer for people to share openly and take risks, (6) embrace creative tension as a source of energy and renewal, and (7) be continuously aware of and interact with their environment. The results of the survey showed a positive move from an organisational blame culture to a culture of success.

Other studies also analysed the application of the LO concept to hospitals and stressed the role of the LO for hospitals [44, 45].

#### The health system as a whole

Sheaff and Pilgrim [46] conducted a study to analyse whether or not characteristics of the LO existed in the NHS, and the organisations constituting the NHS from 1998 to 2006. They conducted a literature review by snowballing references from the founding LO books and published papers. The method was an evaluation based on criteria to analyse to what extent NHS organisations have become like the LO model. They concluded that a LO approach could potentially thrive in a well-funded, unified and politically stable state bureaucracy, as well as a fully autonomous business in a competitive market, or in a single autonomous organisation operating within a competitive but publicly funded health system (a ‘quasi-market’). The other important result coming out of this research was that the NHS as a whole health system cannot act as a global LO, but the organisations that constitute it could be LOs. Friedman and Rigby [47] conducted a descriptive research in the United States about the need to move towards a global learning system based on a review. They emphasised the need for a health system to adopt the characteristics of LO by learning from organisational models that have already proven successful. They argued that learning systems could be created and stressed the importance of an information system to move towards a LO.

Wilkinson et al. [48] compared and contrasted clinical governance, on the one hand, with organisational learning inspired by Senge’s model on the other. The concept of clinical governance was first introduced in the Government’s White Paper for the New NHS (1997) [49]. They found that there is much common ground between the two as well as significant areas of divergence, which require consideration of the notion of a LO. A LO is one which is presented as a desirable notion, with a less well-defined aim and little formal implementation strategy; the LO comes from within the organisation itself with a bottom-up strategy. The introduction of clinical governance was a political act and thus may become associated with scrutiny and judgement from the Government as Trusts endeavour to achieve targets with a top down strategy.

Another study conducted by Timpson et al. [50] in the United Kingdom reviewed the challenges of the NHS’s organisations to embrace tenets of a LO. They used a review on the utility of the concept of LO and concluded that the focus should be on systems that are deliberately designed to facilitate shared learning.

#### Other applications

Other studies have tackled the LO as applied to universities and medical education.

In their study, Rezaee et al. [51] compared two universities of medical sciences in Iran according to the LO concept. They used a LO questionnaire administered to 499 university staff (208 from Shiraz University and 291 from Shiraz University of Medical Sciences). They used the following framework: (1) personnel capability (commitment to constant learning and constant support), (2) common goals (an image of the expected future and practical ways of reaching it), (3) mental images (an image that reflects individual self-images, helping one to take action and form appropriate attitudes and decisions), (4) group learning (teams gather their active energy, capabilities, and insights amounting to more than the sum of their individual skills), and (5) systemic thought (an approach to thinking in which the system takes priority over the individual). They concluded that, because of the mission of universities of medical sciences and educational and research affairs as well as the health and treatment responsibilities, these universities are considered LOs. The application of the LO concept to health education was also discussed by Al-Abri and Al-Hashmi [52] in Saltanat Ouman using the Senge Framework. They concluded that LOs encourage their members to improve their personal skills and qualities. Crites et al. [53] conducted a literature review about LO frameworks. They found seven frameworks (two organisational learning frameworks, the decision-execution cycle framework, the organisational knowledge creation framework, the organisational culture framework, the complex adaptive systems framework, and the diffusion and dissemination of innovation frameworks) and proposed one model that integrated them in the following dimensions: (1) inquiring (acquiring, informing, transforming), (2) deciding (deliberating, decision-taking, evaluating), (3) relating (sharing, cooperating advocating), and (4) interpreting (judging, knowing, formulating). On the other side, Singer et al. [54] developed a short-form questionnaire for the application of LO in health organisations. The questionnaire is a simplified version of the Harvard questionnaire (55 items) based on the model of Garvin [6] and uses data from Veterans Health Administration personnel in the United States (11,336 interviewees via internet). The dimensions that have been used are (1) leadership that reinforce learning, (2) learning processes and practices (experimentation, information collection, analysis, education and training, information transfer), (3) supportive learning environment (psychological safety, appreciation of differences, openness to new ideas, time for reflection). They concluded that it is possible to reliably measure key features of the LO using a 27-item survey adapted from the 55-item Harvard survey.

### Nature of the research

We were also interested in identifying the nature of the research conducted in this field. We defined seven non-exclusive categories for the nature of the research, namely (1) surveys based on scoring systems; (2) review of documents and literature (literature review, reports review, etc.); (3) mixed approaches (literature review or report review completed with interviews); (4) action research; (5) comparative study (where experiences of different settings are analysed according to the LO concept); (6) conceptual and descriptive (discussing a framework and concept definition or describing the LO in an organisation); and (7) diagnostic and assessment (with an objective to determine the level of implementation of LO characteristics. The distribution according to these categories is presented in Table 2.

**Table 2:** Division of sources by nature of the research

References	Scoring system survey and qualitative interviews	Review of documents and literature review	Mixed approaches	Action research	Comparative study	Conceptual and descriptive	Diagnostic and assessment of the learning organisation
[20, 22, 30, 31, 33, 34, 36–39, 45, 51, 54]	13 (48%)						
[25, 26, 29, 42–44, 46, 47, 50, 52, 53]		12(44%)					
[27, 28]			2 (8%)				
[25–27, 30]				4 (15%)			
[42]					1 (4%)		
[28, 29, 44, 45, 47, 48, 50, 52, 53]						9 (33%)	
[20, 22, 32–34, 36–39, 43, 46, 51]							13 (48%)

The above results show that 56% of articles used scoring systems and surveys or mixed approaches to apply the LO concept to the health system. It is also important to mention that only one article used the LO to compare two countries. Thirteen articles used diagnostic and assessment approaches.

### Framework of analysis

Our literature review demonstrated that the bulk of the LO models used are rooted in the original models of Senge [1] and Garvin [6] (Table 3). We have analysed the main dimensions of the 13 identified frameworks, through the lenses of these two frameworks and according to their dimensions.

**Table 3:** Analysis of the learning organisation frameworks according to the Senge [1] and Garvin [41] frameworks

		References in which each framework was used											
	Learning organisation dimensions	[20]	[31]	[51]	[25]	[26]	[37]	[33]	[53]	[43]	[42]	[28]	[54]
(Senge, 1990)	Personal mastery		x	x			x	x		x	x	x	
	Mental models	x		x		x	x						
	Shared vision			x	x		x		x		x		
	Team learning		x	x	x		x	x	x	x	x		
	Systems thinking	x		x			x						
(Garvin, 2008)	Leadership that reinforces learning		x		x	x		x			x		x
	Learning processes and practices	x	x		x			x	x	x	x	x	x
	Supportive learning environment	x	x	x	x	x	x	x	x	X	x		x

By analysing the different dimensions of each of the above frameworks, we found that the majority of the dimensions stress the importance of a learning environment and that learning processes should be embedded in the organisation. Team work and personal mastery were also common dimensions across all these frameworks. Most of the dimensions highlight the importance of the individual level and how personal mastery and individual empowerment can lead to a LO. The leadership role was also a common dimension for these frameworks. This review seems to indicate that there is some convergence towards Garvin’s framework [6].

## Discussion

We have covered a large number of articles and other sources that applied the LO concept to the health context. Most sources highlight the importance and the advantages of applying the LO as a new culture to improve organisational learning for health services. The different applications we reviewed either used a diagnostic tool for LO using qualitative methods, described conceptually the LO, or analysed the impact within an action research approach. Our study also shows that, so far, most of the research on the application of the LO concept in the health context has been undertaken in high-income countries, and mainly in Anglophone countries (Australia, United Kingdom and United States). The application of the LO in the hospital sector has received quite some attention in the reviewed sources. Indeed, characteristics of a LO were found in most well performing hospitals and services. The assessment of the LO in primary health services demonstrated its potential positive impacts to improve quality improvement and performance by improving the organisational learning. The results show that the first line health services, or health centres, have also explored the use of the LO concept and particular interest was expressed in its practical side. Indeed, health centres are the first point of contact for the population within a health system, and therefore satisfaction and the understanding of the changing environment, be it epidemiological or demographical, require learning capacities to better respond to the needs of the population. The above studies showed the existence of LO characteristics through the various approaches at the level of the health system. We also found some links between the LO and the health system when treated as one unit. There is a divergence between the conclusions of several works. On the one hand, one research article concluded that health systems could become learning ones [47] (through a better use of operational data with a partnership among patients, the population and the healthcare services), while another article stated that a health system could not act like an LO, but its component organisations could be LOs, as they are autonomous [46]. Two articles seem insufficient, however, to draw conclusions on whether or not health systems could be learning systems by behaving as one single organisation.

All the frameworks include dimensions related to knowledge and how it is used within the organisation. Research then becomes a tool to document and store knowledge, although not explicitly, in that it could support methodological approaches for collective problem resolution and storing knowledge [4]. Research can also help organisations to learn from the environment, an essential component in almost all the reviewed models.

This review also looked at the assessment process of health organisations using LO diagnostic tools. We have shown that researchers use different frameworks when they apply LO lens in the health sector. Most authors refer to the seminal work by Senge [1, 3] and Garvin [5, 6], but on each occasion, researchers felt the need to adapt the framework to their specific contexts and objectives. In our review, we identified different uses of the LO concept. Some researchers use it to describe existing practices within an action research approach. Others use it to analyse a situation and establish causalities between respecting LO principles and a given performance – we refer to this approach as ‘LO as an analytical framework’. Eventually, we saw some experiences where the LO concept has been used to guide action and change practices within a health service organisation – we can refer to this approach as ‘LO as a prescriptive framework’.

We have also noted that the health research community has so far adopted three main tracks in their empirical investigation – many researchers conduct surveys with individual interviews based on a scoring system (e.g. Likert scale); some use a more ethnographic approach (in-depth interviews); and finally, some researchers, especially when their study target is very large (e.g. the NHS of United Kingdom), mainly review existing documents and publications.

One of the purposes of this review was to inform future attempts to apply the LO lens to health systems in LMICs, particularly with regards to their efforts to make progress towards UHC. Our scoping review confirms that (1) few authors have so far applied the LO lens to a health system as a whole; (2) none of these few ‘health system’ applications were related to LMICs (overall, we found only one LO study in a low-income country in a hospital setting); and (3) no one has so far established an explicit link between LO as a concept and UHC policies and the health system.

These observations should not be misinterpreted: they do not mean that the endeavour to link LO and UHC and other health system strengthening efforts is wrong, instead they indicate that this is an area of study which is yet to be developed. We have noticed the versatility of the LO concept: it can be applied to different levels of the health system, it offers some descriptive, analytical and prescriptive power. Our review has shown that researchers have used different approaches, as far as research methods are concerned, and yet, some common practices also emerged (such as individual polls with scoring systems). What is very clear from our review is that the authors share a common view that the LO concept can be a powerful mode of organisational reform to promote learning within the health sector. This is, for the moment perhaps, more of a hunch than something backed by rigorous evidence, but there is good reason to further explore the power of this concept for health organisations to better achieve their goals.

## Conclusion

Most of the applications of the LO concept in the health sector thus far are related to operational levels and to health facilities in particular. Very little attention has been paid to its application to the health system as a whole, or to health organisations with responsibility for developing policies and strategies (e.g. the central administration of a Ministry of Health). Health systems must be able to adapt and respond to their changing environments. This scoping review showed how the LO concept has been applied to the health sector and has summarised the most important frameworks used for this purpose. It has also documented the different tools used to assess whether or not an organisation is a learning one. We think that this article will help health organisations make choices as to the dimensions they should consider in trying to move towards a LO and integrating knowledge, action and organisational aspects. We intend to use these findings to explore a more holistic model applying LO concurrently at multiple levels within one system. This review reveals a gap in terms of research in this area and a need for more application of the LO concept to the health system as a whole and to the organisations at national level in charge of steering it. Our own hypothesis is that LMICs will not make the progress they want towards UHC without strong autonomous learning capacities. Developing one’s learning capacity may actually be one of the few recommendations valid for all countries as far as UHC is concerned. In this sense, the current momentum for UHC offers a wonderful opportunity to test the power of the LO for enhancing performance of health systems. If researchers support this process, this is a research program which demonstrates potential for further development.

We also encourage further research to explore how to make the LO model more practical and feasible in health systems. Indeed, research needs to be linked to actions being undertaken in health organisations and should bring solutions and serve as a means to store knowledge.

## Abbreviations

DLOQ: dimensions of the learning organisation questionnaire
LMICs: low- and middle-income countries
LO: learning organisation
UHC: universal health coverage

## Footnotes

1 The management literature has its own codes. For instance, Marquardt [4], a leading scholar in this field, provides in his book many examples of private firms where the application of the LO concept was found successful and with an important added value for the performance. Obviously, in the business world, the key test for validating an idea is the extent to which it led to profit – in this respect, a testimony from a senior CEO may be enough. The approach to evidence in the health sector is different and more systematic.

## References

[1] Senge PM (1990). The Fifth Discipline: The Art and Practice of the Learning Organisation.

[2] Garvin DA (1985). Building a learning organisation. Org Dev Trng.

[3] Senge PM (2006). The Fifth Discipline: The Art and Practice of the Learning Organisation.

[4] Marquardt MJ (1996). Building the Learning Organisation.

[5] Garvin DA (2000). Learning in Action: A Guide to Putting the Learning Organisation to Work.

[6] Garvin DA, Edmondson AC, Gino F (2008). Is yours a learning organisation?. Harv Bus Rev.

[7] Ellinger AD, Ellinger AE, Yang B, Howton SW (2002). The relationship between the learning organisation concept and firms' financial performance: An empirical assessment. Hum Resour Dev Q.

[8] Hussein N, Mohamad A, Noordin F, Ishak NA (2014). Learning organisation and its effect on organisational performance and organisational innovativeness: A proposed framework for Malaysian Public Institutions of Higher Education. Procedia Soc Behav Sci..

[9] Wetherington JM, Daniels MK (2013). The relationship between learning organisation dimensions and performance in the nonprofit sector. J Nonprofit Manag.

[10] Rose RC, Kumar N, Pak OG (2009). The effect of organisational learning on organisational commitment, job satisfaction and work performance. J Appl Bus Res.

[11] Rashman L, Withers E, Hartley J (2009). Organisational learning and knowledge in public service organisations: A systematic review of the literature. Int J Manag Rev.

[12] Etienne C, Asamoa-Baah A, Evans DB (2010). Health systems financing: the path to universal coverage.

[13] United Nations General Assembly Sixty-Seventh Session Global Health and Foreign Policy. Adopted 12 December 2012. https://ncdalliance.org/sites/default/files/resource_files/Global%20Health%20and%20Foreign%20Policy%20resolution%202012_67th%20GA. 16 Feb 2017.

[14] Cotlear D, Nagpal S, Smith O, Tandon A, Cortez R. Going universal: how 24 developing countries are implementing universal health coverage from the bottom up. World Bank. 2015. https://openknowledge.worldbank.org/bitstream/handle/10986/22011/9781464806100.pdf?sequence=4&isAllowed=y, 16 Feb 2017.

[15] Kutzin J (2012). Anything goes on the path to universal health coverage? No. Bull World Health Organ.

[16] World Health Organization (2007). Everybody's business--strengthening health systems to improve health outcomes: WHO's framework for action.

[17] Frenk J (2010). The global health system: strengthening national health systems as the next step for global progress. PLoS Med.

[18] Pappaioanou M, Malison M, Wilkins K, Otto B, Goodman RA, Churchill RE (2003). Strengthening capacity in developing countries for evidence-based public health: the data for decision-making project. Soc Sci Med.

[19] Arksey H, O'Malley L (2005). Scoping studies: towards a methodological framework. Int J Soc Res Methodol.

[20] Somunoğlu S, Erdem E, Erdem Ü (2012). A study on determining the perception of learning organisation applications by health sector workers. J Med Syst.

[21] Pelit E, Keleş Y, Çakır M (2010). The perceptions of the employees in hotel business concerning learning organisations: a study on the domestic and international chain hotel businesses. J Organ Admin Sci.

[22] Kelly DR, Lough M, Rushmer R, Wilkinson JE, Greig G, Davies HT (2007). Delivering feedback on learning organisation characteristics using a learning practice inventory. J Eval Clin Pract.

[23] Pedler M, Burgoyne JG, Boydell T (1996). The learning company: a strategy for sustainable development.

[24] Argyris C, Schön DA (1978). Organisational learning: a theory of action perspective.

[25] Schilling L, Dearing JW, Staley P, Harvey P, Fahey L, Kuruppu F (2011). Kaiser Permanente's performance improvement system, Part 4: Creating a learning organisation. Jt Comm J Qual Patient Saf.

[26] Birleson P, Brann P (2006). Reviewing the learning organisation model in a child and adolescent mental health service. Aust Health Rev.

[27] Birleson P (1998). Learning organisations: a suitable model for improving mental health services?. Aust N Z J Psychiatry.

[28] Cantle F (2000). What is a ‘learning organisation’ in general practice? A case study. Health Serv Manage Res.

[29] O'Connor N, Kotze B (2008). ‘Learning Organisations’: a clinician's primer. Australas Psychiatry..

[30] Bunniss S, Gray F, Kelly D (2012). Collective learning, change and improvement in health care: trialling a facilitated learning initiative with general practice teams. J Eval Clin Pract.

[31] Leufvén M, Vitrakoti R, Bergström A, Kc A, Målqvist M (2015). Dimensions of Learning Organisations Questionnaire (DLOQ) in a low-resource health care setting in Nepal. Health Res Policy Syst..

[32] Marsick VJ, Watkins KE (2003). Demonstrating the value of an organisation's learning culture: the dimensions of the learning organisation questionnaire. Adv Dev Hum Resour.

[33] Mohebbifar R, Hashemi HJ, Rajaee R, Najafi M, Etedal MG (2015). Learning organisation profile of educational hospitals in Iran: practice of organisational interlocking systems. Glob J Health Sci.

[34] Estrada N (2009). Exploring perceptions of a learning organisation by RNs and relationship to EBP beliefs and implementation in the acute care setting. Worldviews Evid Based Nurs.

[35] Watkins KE, Marsick VJ (1993). Sculpting the learning organisation: lessons in the art and science of systemic change.

[36] Dias C, Escoval A (2015). Hospitals as learning organisations: fostering innovation through interactive learning. Qual Manag Health Care.

[37] Oudejans SCC, Schippers GM, Schramade MH, Koeter MWJ, Van den Brink W (2011). Measuring the learning capacity of organisations: development and factor analysis of the Questionnaire for Learning Organisations. BMJ Qual Saf.

[38] Jeong SH, Lee T, Kim IS, Lee MH, Kim MJ (2007). The effect of nurses’ use of the principles of learning organisation on organisational effectiveness. J Adv Nurs.

[39] Ugurluoglu O, Ugurluoglu Aldogan E, Dilmac E (2013). The impact of managers' perceptions of learning organisations on innovation in healthcare: sample of Turkey. Int J Health Plann Manage.

[40] Watkins KE, Marsick VJ (1997). Dimensions of the Learning Organisation Questionnaire.

[41] Garvin DA (1993). Building a learning organisation. Harv Bus Rev.

[42] Vassalou L (2001). The learning organisation in health-care services: theory and practice. J Eur Ind Train.

[43] Rowley SD (2006). The journey of a teaching hospital to become a learning organisation. Aust Health Rev.

[44] Cathon DE (2000). The learning organisation: adapted from the fifth discipline by Peter Senge. Hosp Mater Manage Q.

[45] Tsai Y (2014). Learning organisations, internal marketing, and organisational commitment in hospitals. BMC Health Serv Res..

[46] Sheaff R, Pilgrim D (2006). Can learning organisations survive in the newer NHS. Implement Sci..

[47] Friedman C, Rigby M (2013). Conceptualizing and creating a global learning health system. Int J Med Inform.

[48] Wilkinson JE, Rushmer RK, Davies HT (2004). Clinical governance and the learning organisation. J Nurs Manag.

[49] Department of Health (1997) The New NHS Modern and Dependable. The Stationery Office, London.https://www.gov.uk/government/uploads/system/uploads/attachment_data/file/266003/newnhs.pdf, 16 Feb 2017.

[50] Timpson J (1998). The NHS, as a learning organisation: aspirations beyond the rainbow?. J Nurs Manag.

[51] Rezaee R, Yazdani Z, Shokrpour N (2014). Comparison of learning organisation indicators in 2 universities in Shiraz as viewed by the personnel. Health Care Manag.

[52] Al-Abri RK, Al-Hashmi IS (2007). The learning organisation and health care education. Sultan Qaboos Univ Med J.

[53] Crites GE, McNamara MC, Akl EA, Richardson WS, Umscheid CA, Nishikawa J (2009). Evidence in the learning organisation. Health Res Policy Syst..

[54] Singer SJ, Moore SC, Meterko M, Williams S (2012). Development of a short-form learning organisation survey: the LOS-27. Med Care Res Rev.

